# The Ebola virus – going beyond the bleeding edge

**DOI:** 10.1099/jmm.0.001998

**Published:** 2025-07-03

**Authors:** Saadiya K Umar, Mathew A Diggle

**Affiliations:** 1 Provincial Laboratory for Public Health (ProvLab), Alberta Precision Laboratories (APL), Alberta Health Services, Edmonton, Alberta T6G 2J2, Canada; 2 University of Alberta, Edmonton Alberta, T6G 1C9, Canada

**Keywords:** Ebola virus (EBV), Ebola virus genus, mortality, outbreak, viral hemorrhagic fever, zoonosis

## Abstract

An erratum of this article has been published full details can be found at https://doi.org/10.1099/jmm.0.002052

The Ebola virus (EBV) genus is responsible for severe viral haemorrhagic illness caused by a group of viruses belonging to the Filoviridae family. Five species have been identified as causative agents for Ebola virus disease (EVD). The EBV (Zaire) strain is the most predominant organism involved in recorded EVD outbreaks and has been reported as the most virulent. EVD was first identified in the Democratic Republic of Congo in 1976 and has occurred in sporadic outbreaks over the last few decades with the most recent episode in Uganda over the period September 2022–January 2023. EVD is zoonotic in nature with bats as reservoir host and humans become infected via a spillover event from contact with infected animals. EVD is transmitted through contact with infected bodily fluids. It is considered fatal with a high mortality and high infectivity rate. Treatment is generally supportive with the availability of intravenous fluids and medicines. Research into vaccine development is ongoing. EVD is a particular public health concern given the potential for global spread during an outbreak.

## Historical Perspective

Ebola virus disease (EVD) first emerged in 1976 with outbreaks in Nzara, Sudan (now South Sudan) and Yambuku, Zaire (now Democratic Republic of Congo). The virus was named after the Ebola River near Yambuku in the Democratic Republic of Congo [1,2]. The first outbreak of EVD in Nzara, Sudan began when a cloth room worker at the Nzara Cotton Manufacturing Factory, identified as YuG, fell ill with a haemorrhagic febrile disease. YuG experienced severe fever, headaches and chest pains; as his condition worsened, he was brought to the Nzara hospital, where he developed gastrointestinal bleeding and died just 9 days after the onset of symptoms (ref). Following YuG’s death, the virus spread to other factory workers and their families. The epidemiologically most significant case was PG, who worked alongside YuG. PG became ill and, again as with YuG, died just 9 days after the onset of symptoms, with 69% of all EVD cases in Nzara traced back to him [1]. The Yambuku outbreak began when the village school’s headmaster, Mabalo Lokela, fell ill after returning from a trip to Northern Zaire. Initially misdiagnosed with malaria, Lokela’s condition worsened, and he succumbed to the disease on 8 September, just 14 days after symptom onset [1]

The EVD strains are named based on a standardized system that incorporates several key factors and follows several principles, namely, species names based on the geographical region they were first identified. Below the species level, strains and isolates are named using a detailed format which includes virus name, isolation host suffix, country of sampling, year of sampling, genetic variant designation and isolate designation. Finally, phylogenetics is used to classify and name individual isolates; for example, viruses less than 30% genomic differences from the type of strain belong to the same species, while those with more than 10% differences within a species may be considered new strains [3].

The mode of initial transmission is zoonotic in nature via wild animals and subsequently spreads via human-to-human transmission. There had been no cases or outbreaks recorded between 1979 and 1994. However, since that time, sporadic outbreaks have occurred with increasing frequency. The outbreak in West Africa encountered between March 2014 and June 2016 was the largest to date with ~28,000 cases and ~11,000 deaths [4,5].

## Clinical presentation

The clinical features of EVD are generally non-specific and can mimic common causes of febrile illness in returning travellers, such as malaria, typhoid and dengue fever. Incubation periods range from 2 to 21 days, and illness progresses from ‘dry’ symptoms (fever, aches, fatigue) to ‘wet’ gastrointestinal symptoms (diarrhoea and vomiting). The spectrum of clinical features is often systematic with EVD involving all organs manifesting as headaches, dizziness, ataxia, memory loss, encephalitis, paresthesia, uveitis, hearing loss, tinnitus, pulmonary oedema, hepatic dysfunction, renal dysfunction [6], circulatory/cardiovascular, reproductive system, osteoarticular and musculoskeletal manifestations. Some respiratory symptoms, hiccups, conjunctival injection and macropapular rash are also often reported. Haemorrhagic manifestations are observed in half of the infected persons, with no clear correlation between bleeding and disease severity. The presence of manifestations other than haemorrhaging had favoured the use of the term EVD instead of Ebola haemorrhagic fever. It is well established that a person infected with EVD is not contagious until the appearance of symptoms [6]. The average EVD case fatality rate is around 50%, and case fatality rates varied from 25% to 90% during previous outbreaks. Case fatality rates of EBV infections vary from no fatalities (0% case fatality rate) in Reston virus and Tai Forest virus infections to reaching up to 90% in EBV infection [7].

## Microbial characteristics

### Phenotypic

**Fig. 1.**
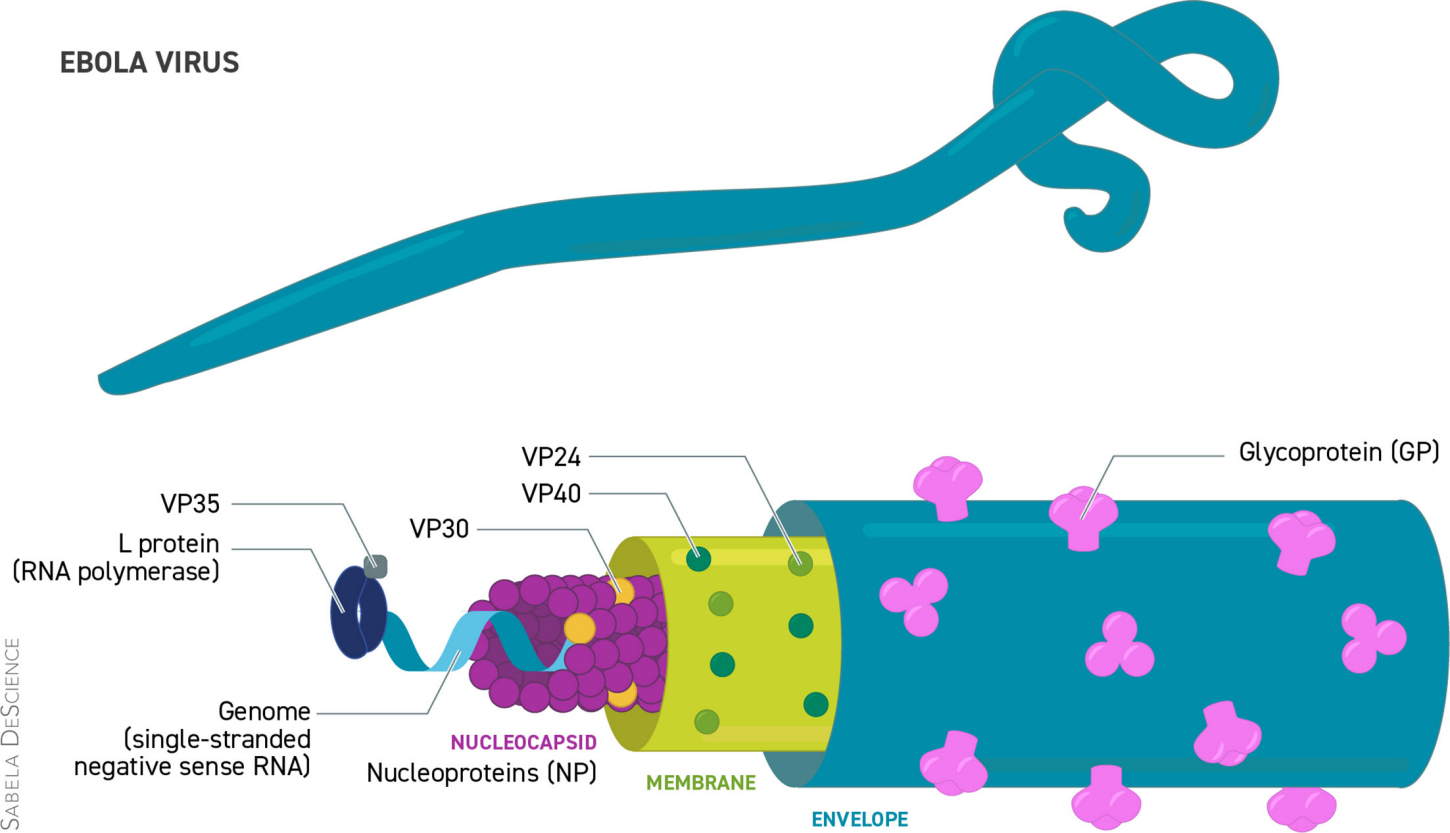
Structure of the Ebola Virus.

EBV (Fig. 1) belongs to the family Filoviridae (the same family as the Marburg virus, genus EBV). There are five EBV species, of which four are associated with human infections (Bundibugyo, Sudan, Tai Forest and EBV) [8]. Reston virus has not been associated with disease; therefore, it is not identified as a human pathogen. In 2018, a sixth new strain, the Bombali virus, was discovered in molossid bats in the Bombali area in North Sierra Leone, which has no recorded human infections to date [9].

### Genotypic

EBV strain is an enveloped filamentous virus with a helical nucleocapsid measuring 400–14,000 nm. It has a linear 19 kb length, non-segmented, single-stranded negative-sense R RNA genome expressing seven structural proteins and several non-structural proteins from seven genes [10]. The seven genes encode nucleoproteins, glycoproteins, RNA-dependent RNA polymerase large proteins and four viral particles: VP24 RNA complex-associated proteins, VP30 transcriptional activators, VP35 polymerase cofactors and VP40 matrix proteins. Thus, four proteins, nucleoproteins, VP35, VP30 and large proteins, are associated with the viral RNA genome. Two proteins, VP24 and VP35, antagonize the type I IFN responses, favouring innate immune evasion [11].

## Clinical diagnosis, laboratory confirmation and safety

### Clinical diagnosis

A case definition has been developed to support surveillance programmes and as a screening tool to identify suspected cases and diagnostic evaluation. Case definitions are also often refined to reflect clinical and epidemiological features associated with a particular outbreak context. This is to enhance balanced sensitivity and specificity in order not to miss individuals requiring treatment and potentially spreading the disease and not to overburden healthcare systems and the limited laboratory capacity needed to confirm a clinical suspicion [12]. This is quite challenging as case definitions rely mainly on clinical signs and risk factors reported by individuals. As such, there is no uniform EVD case definition that is accepted as a global standard. However, international stakeholder organizations such as the World Health Organization (WHO) have provided frameworks as case definitions outlined in the Integrated Disease Surveillance and Response guidelines 2010 to support public health surveillance and enact effective response to EV outbreaks. Public health authorities utilize these standard case definitions to optimize surveillance and notification of EVD, particularly before an outbreak has been identified [13]. There are also nationally developed frameworks within specific countries such as the UK and Canada. For management of outbreaks, the WHO has provided frameworks for suspected, probable, confirmed and discarded cases for identification, management and surveillance purposes [14,15]:

Suspected case

- any person, alive or dead, suffering or having suffered from sudden onset of high fever, and had contact with
  - a suspected, probable or confirmed EVD case, or a dead or sick animal; **OR**
- any person with sudden onset of high fever, **AND** at least three of the following symptoms: headache, lethargy, anorexia/loss of appetite, aching muscles or joints, stomach pain, difficulty swallowing, vomiting, difficulty breathing, diarrhoea, hiccups **OR**
- any person with inexplicable bleeding O**R**
- any sudden, inexplicable death.

Probable case

- any suspected case evaluated by a clinician **OR**
- any deceased suspected case (where it has not been possible to collect specimens for laboratory confirmation) having an epidemiological link with a confirmed case.

Confirmed case

- any suspected or probable case with a positive laboratory result (detection of EBV by reverse transcription PCR, or detection of IgM antibodies directed against EBV).

Discarded case.

- any suspected or probable case with a negative laboratory result (showing no specific antibodies, RNA or specific detectable antigens).

## Laboratory confirmation

The gold standard for confirmatory testing involves isolation of the virus, detection of particle components (antigen or nucleic acid-RNA) and/or demonstration of antibodies against the virus or a PCR-positive result by two molecular targets (Table 1). Single PCR from blood taken >72 h post-symptom onset is sufficient for diagnostic testing and confirmation of the disease [14]. Viral antigen and nucleic acid detection in blood is possible only in symptomatic patients from day 3 after the onset of symptoms, which peaks around day 7 post-symptom onset, and can remain high throughout the course of the disease in fatal cases [16]. Measurement of viral load is also critical as several clinical studies have shown a strong correlation between viremia titre and EVD fatality, with high blood viral load (≥106 copies/ml) being a strong predictor of fatal outcome. A whole blood sample is recommended for virological diagnosis. In general, samples for EBV testing are also tested for malaria (rapid antigen test, PCR) and other routine haematology (e.g. CBC+D) and chemistry tests (e.g. electrolytes, LFTs and serum creatinine) [17]. Additionally, the recipient reference laboratory may also run other haemorrhagic fever panels for travel-related viral infections such as Congo Crimean Haemorrhagic Fever, Rift Valley Fever, Junin, Lassa and Marburg. Appropriate testing is often dictated by the phase of the illness (Table 1).

**Table 1.** EVD timeline of infectious phases and appropriate tests

Timeline of infection	Diagnostic tests	Additional information
Within a few days after symptom onset	**Antibody-capture Enzyme-Linked Immunosorbent Assay (IgM**) **Antigen-capture detection tests** (Demonstration in tissue by immunohistochemical or immunofluorescent techniques) **Serum neutralization test** **Reverse-transcriptase PCR assay** **Electron microscopy** **Virus isolation by cell culture** (blood, serum, tissue, urine and throat secretions)	PCR is the most common diagnostic method, ability to detect low levels of EV. Molecular targets used are nucleoprotein, glycoprotein and large protein genes.
Later in the disease course or after recovery	**IgM and IgG antibodies** (by country’s National Reference Laboratory, Centre for Disease Control, or approved WHO collaboration centre)	Demonstration of IgM +with rising IgG or decreasing IgM with four times rise in IgG titre in paired successive samples.
Retrospectively in deceased patients	**Immunohistochemistry** **PCR** (blood, serum, oral fluid, tissue) **Virus Isolation (performed at the National Reference Laboratory**)	**Current WHO recommended tests include:** Automated or semi-automated nucleic acid test for routine diagnostic management. Rapid antigen detection tests for use in remote settings where nucleic acid tests are not readily available.

WHO, World Health Organization.

## Laboratory safety

All members of the genus EBV are Risk Group 4 human and animal pathogens, classified as Biosafety Level 4/Containment Level 4 and a security-sensitive biological agent. In many jurisdictions, there are additional security requirements, such as obtaining a Human Pathogens and Toxins Act Security Clearance, for work involving security sensitive biological agents. Sample collection and processing must be done by trained healthcare workers, with the greatest attention to biosafety measures including additional personal protection equipment: gloves, masks, goggles (preferably with anti-fog visor) and waterproof apron (preferably disposable) [18]. Clinical samples collected from patients are considered an extreme biohazard risk; laboratory testing on non-inactivated samples should be conducted under maximum biological containment conditions. All activities with infectious material should be conducted in a biological containment unit, e.g. closed container centrifugation, closed system blood cultures preparation and sub-culturing should only be performed if it is essential to patient care as it has the potential to generate aerosols. In addition, in many jurisdictions, all biological specimens should be packaged using the triple packaging system when transported nationally and internationally along with an activated emergency response activation plan or equivalent [19]. An organized emergency response activation plan would be activated prior to transporting suspect EBV samples from one facility to another. If there is an incident while transporting the samples, there is an emergency response activation plan team or equivalent to it in place to respond to the biohazardous nature of the potential contamination [14].

## Treatment and resistance

Similar to the diagnosis of EVD, treatment guidelines are provided by international stakeholder organizations, such as the WHO’s Ebola Treatment Guideline, August 2022 [20]. It is the first guideline for EVD therapeutics, based on systematic reviews and meta-analysis of Randomised Controlled Trials (RCTs) conducted during outbreaks in the Democratic Republic of Congo. Guiding management approaches include:

### Supportive care

This represents clinical care guidance that is a package of interventions that outline early diagnosis and optimized supportive care – these include relevant tests to administer, managing pain, ensuring electrolyte balance, rehydration and nutrition, as well as management of co-infections and other approaches that place the patient on the best path to recovery and, as a consequence, significantly improving survival [21].

### Therapeutic care

There are strong recommendations for the use of two monoclonal antibodies mAb114 (Ansuvimab; Ebanga) and REGN-EB3 (Inmazeb) as treatment agents. Atoltivimab/maftivimab/odesivimab, sold under the brand name Inmazeb, is a fixed-dose combination of three monoclonal antibodies, and Ansuvimab, sold under the brand name Ebanga, is a monoclonal antibody medication, both used for the treatment of EVD. These agents have demonstrated clear benefits and development of a new standard of care for patients, which can be used for all patients confirmed with the disease [22]. The agents have demonstrated efficacy in a wide range of population subgroups including older people, pregnant and breastfeeding women, children and newborns of mothers with confirmed disease within 7 days of delivery. Patients should receive recommended neutralizing monoclonal antibodies as soon as possible after laboratory confirmation of diagnosis. Other alternative agents such as ZMapp, which is an experimental biopharmaceutical medication consisting of a combination of three chimeric monoclonal antibodies that target the EV, are no longer recommended due to the availability of more effective treatments and remdesivir, which showed promise in preclinical studies, although it was ultimately found to be less effective at reducing mortality than antibody-based therapies during outbreaks. Of note, all these recommendations only apply to EVD caused by EBV. For the other strains, there are no therapeutic agents of benefit to date [22].

### Transmission

**Fig. 2.**
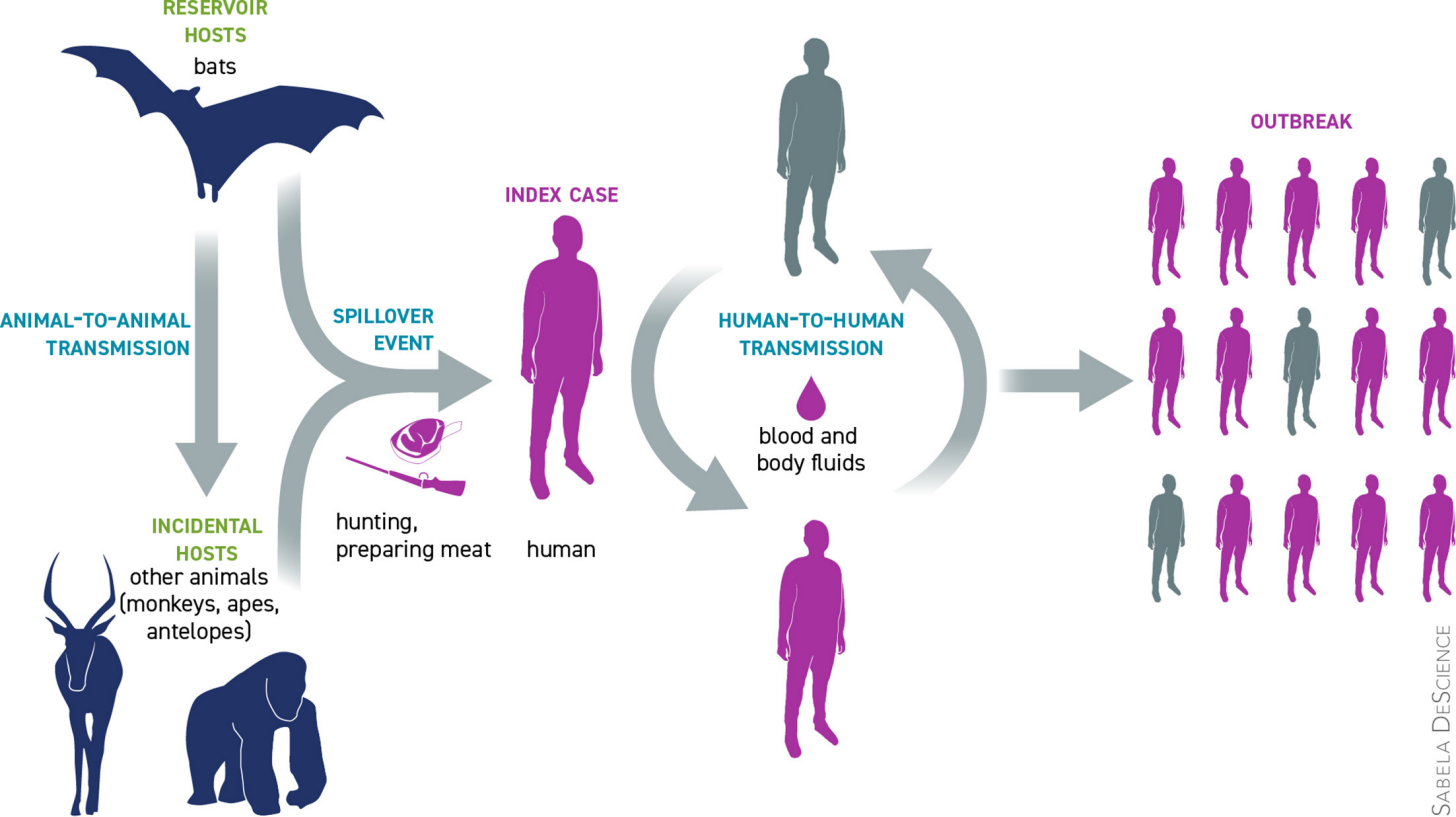
Transmission of Ebola Virus.

The natural reservoir host(s) of EBV are forest-dwelling fruit bats of the Pteropodidae family in Africa (Fig. 2). Other animals, in particular non-human primates, have been identified as incidental hosts (not reservoirs) based on multiple data, although EBV has neither been isolated from nor has a near-complete EBOV genome been detected in any wild animal [23].

Disease occurs in outbreaks which can be traced back to a single spillover introduction of EBV into the human population from an unidentified reservoir by unknown mechanisms. Transmission can be animal to animal, animal to humans (both in a spillover event, direct transmission from reservoir host) and human to human. Bats carrying the virus can transmit it to other animals (monkeys, apes, antelopes), as well as to humans. This, as mentioned, is known as a ‘spillover event’ that occurs when an animal or human becomes infected with EBV following contact with the reservoir host either through hunting or preparing the animal's meat for eating [24]. Once the first human transmission occurs (referred to as ‘index case’), human-to-human transmission of the virus is maintained through contact with blood and body fluids of sick persons or dead bodies of those who have died of EVD. Patients can transmit EBV once symptomatic, as well as in postmortem state. Incubation period can be between 2 and 21 days with the virus detectable in blood at >3 days post-infection [24].

### Infection

Most human cells can become infected, but mononuclear phagocytes (e.g. Kupffer cells in the liver, macrophages, microglia and dendritic cells) are primary EBV targets. Upon infection, the virus affects the host blood coagulation and immune defence system and leads to a severe immunosuppressive state. EBV particles enter the body through dermal injuries (microscopic or macroscopic wounds) or via direct contact with mucosal membranes. Cellular entry is through endocytosis and replication occurs in the cytoplasm. The primary target cells, which are infected macrophages and dendritic cells, migrate to regional lymph nodes while producing progeny virions. This facilitates further virus dissemination and migration via regional lymph nodes to the liver, spleen and various parts of the body. Through suppression of intrinsic, innate and adaptive immune responses, the systemic distribution of progeny virions and the infection of secondary target cells occur in all organs [25].

### Virulence factors

The main EBV proteins considered responsible for its virulence are VP35, VP24 and glycoproteins. VP35 and VP24 function as an IFN antagonist (Fig. 1). VP35 blocks host type I IFN-α/β induction and the phosphorylation of double-stranded RNA-activated protein kinase R that mediates cellular antiviral responses, while VP24 interferes with type I IFN signalling cascade by blocking karyopherin α-mediated nuclear trans localization of phosphorylated STAT1 homodimers or STAT1–STAT2 heterodimers. This restriction of STAT nuclear translocalization by VP24 leads to reduced transcriptional activation of IFN-stimulated genes, preventing the antiviral state in the host cells. The glycoproteins interact with host toll-like receptor 4, leading to the activation of pro-inflammatory responses via the NF-κB pathway [26].

## Epidemiology, prevention and risk groups

### Epidemiology

Since the emergence of EBV in 1976 in the Democratic Republic of the Congo (then Zaire), there have been 38 country-specific outbreaks that originated in Gabon, Guinea, the Democratic Republic of the Congo [27]. The total estimated EVD deaths from 1976 to 2020 were ~15,000. The largest outbreak was in West Africa between March 2014 and June 2016 with ~28,000/~11,000 recorded cases/fatalities. This began in February 2014 in Guinea, with the most prevalent EVD outbreak recorded in history which spread to Liberia, Sierra Leone, Nigeria, Senegal, Spain and the USA. It is by far the largest so far and most deadly caused by the EBOV strain. The most recent outbreak occurred in Uganda from September 2022 to January 2023 and originated from Mubende district in central Uganda, caused by the *Sudan ebolavirus*. The outbreak was declared over on 11 January 2023, with 164 confirmed cases and 55 confirmed deaths. The average EVD case fatality rate was ~50% with case fatality rate variation from 25% to 90% in past outbreaks, depending on circumstances and the response. Of the EBV *spp*, EBOV, species *Zaire ebolavirus* (the cause of EVD), is the most lethal, with case fatality rates between 70% and 90% if left untreated. EBV is responsible for the most recorded EVD outbreaks, including the largest EVD outbreaks in history, the 2014–2016 West Africa outbreak and the 2018 outbreak in the eastern Democratic Republic of the Congo, where over 32,000 people were infected, and more than 13,600 deaths were reported. Importation of EVD to other countries and regions of the world by an infected traveller from an outbreak area is a recognized risk with the potential for spread into the population. During the 2014–2016 EVD outbreak in West Africa, 11 people were treated for EVD in the USA, and 2 died. Nine of these cases were imported into the USA. Two were domestic healthcare workers who were infected while caring for the first travel-associated EVD case diagnosed in the USA. Both healthcare workers recovered [28,29].

### Prevention

Early recognition and basic infection control strategies are key to controlling EBV spread. When approaching a suspected case, the use of appropriate personal protection equipment [gloves, masks, goggles (preferably with anti-fog visor) and waterproof apron (preferably disposable)] is paramount with additional precautions to ensure separation and isolation of the patient from others [19]. It is important to remember the following three I’s (identify, isolate and inform):

**Identify** and assess the patient for international travel (look for epidemiologic risk factors in all travellers returning from areas with an active EBV outbreak.), contact with someone with EBV in the last 21 days and compatible symptoms. If the patient evaluation indicates possible EBV infection (consistent relevant exposure or symptoms present), it is important to act and isolate the patient and inform appropriate others [14].

**Isolate** the patient in a private room and begin immediate infection prevention and control measures [13] (follow the WHO Infection prevention and control guideline for EBV and Marburg Virus, August 2023):

- Isolate the patient in a single room with a private bathroom or covered, bedside commode.
- Adhere to infection prevention and control procedures to prevent transmission through direct or indirect contact, including wearing appropriate personal protection equipment and using dedicated equipment.
- Use only essential healthcare workers trained in their designated roles for patient care and keep a log of everyone who enters and leaves the patient’s room.
- Perform only necessary tests and procedures and avoid aerosol-generating procedures.

**Inform** other healthcare personnel, facility and public health authorities (local, regional and national) for EBV testing and further control measures. A single negative result using reverse-transcriptase PCR testing, at 48–72 h apart, is required in most jurisdictions to discharge a suspected case [14].

### Vaccination

Vaccination using ERVEBO^®^ (Ebola Zaire live attenuated vaccine, also known as V920, rVSVΔG-ZEBOV-GP or rVSV-ZEBOV) is a replication-competent, live, attenuated recombinant vesicular stomatitis virus (rVSV) vaccine manufactured by Merck approved for the prevention of disease caused by EBV in individuals 12 months of age and older as a single dose administration. In many jurisdictions, it is recommended by expert advisory groups on immunization as part of a broader set of EBV outbreak response tools [30].

ERVEBO was licensed in November 2019 by the European Medicines Agency and prequalified by WHO. The United States Food and Drug Administration licensed the vaccine in December 2019. Burundi, Central African Republic, the Democratic Republic of the Congo, Ghana, Guinea, Rwanda, Uganda and Zambia have also approved the vaccine for use. ERVEBO does not provide protection against other species of EBV or Marburg virus. In May 2020, the European Medicines Agency recommended a second new vaccine delivered in two doses called Zabdeno (Ad26.ZEBOV) and Mvabea (MVA-BN-Filo) for individuals 1 year and older: Zabdeno is administered first and Mvabea is given approximately 8 weeks later as a second dose. This prophylactic 2-dose regimen is, therefore, not suitable for an outbreak response where immediate protection is necessary [30,31].

### Risk groups classified into high and low risk

1. **High Risk**:
  1. Healthcare providers who do not use proper infection control while caring for patients with EBV, including working in a laboratory where human specimens are handled without wearing appropriate personal protection equipment.
  2. Unprotected direct contact with body fluids of a person infected with EBV or who has died of EBV.
  3. Contact with infected fruit bats and primates (apes and monkeys).
2. **Low Risk**:
  1. People with a travel history to countries with widespread transmission or exposure to a person with EBV.
  2. Contact with semen from a man who has recovered from EBV (e.g. by having oral, vaginal or anal sex).

### Open questions

1. Natural Reservoir and Ecology – The complete transmission cycle in nature remains unclear, including how the virus circulates between reservoir species. What drives viral spillover events from animals to humans?
2. Guidelines – What are the current standards for clinical evaluation and application of public health measures?
3. Pathogenesis and Host Response – what questions remain unanswered regarding the evasion and persistence of EVD?
4. Vaccinations – What current knowledge do we have on the duration of immune protection after natural infection or vaccination?
5. Viral Evolution – How quickly does the EBV evolve during an outbreak? Does it have the capacity to adapt to new hosts?

## Abbreviations

EBV: Ebola virus
EVD: Ebola virus disease
RCTs: randomised controlled trials
WHO: World Health Organization
