# Erratum: The Ebola virus – going beyond the bleeding edge

**DOI:** 10.1099/jmm.0.002052

**Published:** 2025-08-19

**Authors:** Saadiya K. Umar, Mathew A. Diggle

**Affiliations:** 1 Provincial Laboratory for Public Health (ProvLab), Alberta Precision Laboratories (APL), Alberta Health Services, Edmonton, Alberta T6G 2J2, Canada; 2 University of Alberta, Edmonton, Alberta, T6G 1C9, Canada

The original version of this article missed a citation in the following sentence:

“YuG experienced severe fever, headaches and chest pains; as his condition worsened, he was brought to the Nzara hospital, where he developed gastrointestinal bleeding and died just 9 days after the onset of symptoms.’’

The following reference should have been cited:

(1) “**World Health Organization (WHO**) Ebola virus disease, Fact sheet N°103, September 2014; 2014 https://web.archive.org/web/20141214011751/https://www.who.int/mediacentre/factsheets/fs103/en/

The Microbiology Society apologises for any inconvenience caused. The original version of this article has been updated to reflect this correction.

